# Doses of Calomel in Days of Yore

**Published:** 1826-07

**Authors:** 


25. *Doses of Calomel in Days of Yore.* In the seventh volume of Haller's Dissertations, the curious reader will find an important paper on the use of calomel in various diseases, by Michaelis Alberti, in which he gives a learned history of this potent medicine, from the Arabian physicians down to Paracelsus, and thence to his own time, 1745. In this dissertation it will be seen that Helwichius gave calomel in doses of five scruples to two patients. To a third he gave 72 grains, which affected the mouth for a fortnight. Neuterus gave calomel, at first, in the dose of 15 grains, second dose a scruple, third dose, half a drachm—fourth dose, a drachm, at which dose he continued till ptyalism was raised. The intervals between the doses are not mentioned. Medelius advises the dose of calomel not to be raised beyond a scruple. From this and many other sources of information it will be seen that there is nothing new under the sun.

